# 10351 Antibiotic Use for Respiratory Syncytial Virus in the Middle East: A Surveillance Study in Hospitalized Jordanian Children

**DOI:** 10.1017/cts.2021.476

**Published:** 2021-03-30

**Authors:** Danielle A. Rankin, Nikhil K. Khankari, Zaid Haddadin, Olla Hamdan, Samir Faouri, Asem Shehabi, John V. Williams, Najwa Khuri-Bulos, Natasha B. Halasa

**Affiliations:** 1 Vanderbilt University Medical Center; 2 Al Bashir Hospital; 3 Jordan University; 4 University of Pittsburgh School of Medicine

## Abstract

ABSTRACT IMPACT: Antibiotic stewardship guidelines should consider the barriers clinicians in low- and middle-income countries face due to limited biomarkers for determining the etiologic pathogen for viral infections like respiratory syncytial virus (RSV) that have a similar presentation to bacterial infections. OBJECTIVES/GOALS: We aimed to evaluate antibiotic administration practices in children who were hospitalized at a government-run hospital in Amman, Jordan, where point-of-care testing is limited. We hypothesized those with RSV are more likely to be administered antibiotics during their hospitalization than children without RSV. METHODS/STUDY POPULATION: We conducted a cross-sectional cohort study in Jordanian children hospitalized with history of acute respiratory symptoms and/or fever from 2010 to 2013. Admitting diagnoses were dichotomized into suspected viral- (e.g., bronchiolitis) and bacterial-like infection (e.g., sepsis, pneumonia). Stratifying by sex, we performed a polytomous logistic regression adjusting for age, underlying medical condition, maternal education, and region of residence to estimate prevalence odds ratios (PORs) and 95% confidence intervals for macrolides, broad-, and narrow-spectrum antibiotics during hospitalization. Sensitivity and specificity of admission diagnoses and laboratory results were compared. RESULTS/ANTICIPATED RESULTS: Children with a suspected viral-like admission diagnosis, compared to those with suspected bacterial-like, were 89% less likely to be administered a narrow-spectrum antibiotic (POR: 0.11; p<0.001). There were slight differences by sex with males having a lower prevalence than females of narrow-spectrum or broad-spectrum antibiotic administration; but they had a higher prevalence of macrolide administration. Overall, children with RSV had a 30% probability (sensitivity) of being assigned to a suspected viral infection; whereas RSV-negative children had an 85% probability (specificity) of being assigned to a suspected bacterial infection. DISCUSSION/SIGNIFICANCE OF FINDINGS: Children with a suspected viral-like infection were less likely to receive an antibiotic; however, when evaluating the accuracy of admission diagnosis to RSV-laboratory results there were considerable misclassifications. These results highlight the need for developing antibiotic interventions for Jordan and the rest of the Middle East.

